# Development of a Layer Made of Natural Fibers to Improve the Ecological Performance of the Face Mask Type II

**DOI:** 10.3390/ma16165668

**Published:** 2023-08-17

**Authors:** Jerzy Mańkowski, Małgorzata Zimniewska, Weronika Gieparda, Barbara Romanowska, Anna Kicińska-Jakubowska, Jacek Kołodziej, Joanna Foksowicz-Flaczyk, Szymon Rojewski, Krzysztof Bujnowicz, Patrycja Przybylska, Edyta Kwiatkowska, MD Masud Alam, Wanda Różańska, Aleksandra Wawro, Elżbieta Hołderna-Kędzia

**Affiliations:** Institute of Natural Fibers and Medicinal Plants National Research Institute, Wojska Polskiego 71B, 60-630 Poznan, Poland; jerzy.mankowski@iwnirz.pl (J.M.); malgorzata.zimniewska@iwnirz.pl (M.Z.); weronika.gieparda@iwnirz.pl (W.G.); anna.jakubowska@iwnirz.pl (A.K.-J.); jacek.kolodziej@iwnirz.pl (J.K.); joanna.flaczyk@iwnirz.pl (J.F.-F.); szymon.rojewski@iwnirz.pl (S.R.); krzysztof.bujnowicz@iwnirz.pl (K.B.); patrycja.przybylska@iwnirz.pl (P.P.); edyta.kwiatkowska@iwnirz.pl (E.K.); masud.alam@iwnirz.pl (M.M.A.); wanda.rozanska@iwnirz.pl (W.R.); aleksandra.wawro@iwnirz.pl (A.W.); elzbieta.kedzia@iwnirz.pl (E.H.-K.)

**Keywords:** face mask type II, flax fibers, cotton, nonwoven, polymer film, ecological aspects

## Abstract

The aim of this study was to develop a natural nonwoven layer made of cottonized bleached flax and cotton fibers which is suitable to replace one of the three polypropylene layers of face mask type II in order to reduce non-biodegradable waste production and limit the negative impact of used masks on the environment. The work focused on the design of a nonwoven structure based on properly blending cotton and flax fibers as well as ensuring the cover factor, which can support the mask’s barrier properties against air dust particles and does not make breathing difficult. Additionally, a biodegradable film was developed to connect the nonwoven layer with the other polypropylene filtering layers. The effectiveness of the biodeterioration of the flax/cotton nonwoven was evaluated based on a test of the susceptibility of materials to the action of soil microorganisms. The flax/cotton nonwoven layer was tested in terms of mechanical, physical, and biophysical properties, and an analysis of the covering of the nonwoven surface with fibers was conducted as well. The results confirmed that the structure of flax/cotton nonwovens is suitable to replace the nondegradable polypropylene layer of the face mask type II to improve its environmental performance.

## 1. Introduction

On 5 May 2023, the Director General of the World Health Organization (WHO) announced the end of the COVID-19 pandemic as a global health emergency. He noted that: “The worst thing any country could do now is to use this news as a reason to let down its guard, to dismantle the systems it has built, or to send the message to its people that COVID-19 is nothing to worry about”. Therefore, the WHO has published the fourth edition of the Global Strategic Preparedness and Response Plan for COVID-19, which outlines five main areas of action in the event of a pandemic: collaborative surveillance, community protection, safe and scalable care, access to countermeasures, and emergency coordination [1].

The global situation related to the spread of the COVID-19 pandemic has forced users to pay attention to the use of personal protective equipment, including protective masks, particularly health care facilities, such as hospitals [2].

The COVID-19 pandemic situation proved that any dangerous viruses, pathogenic bacteria or fungi can spread around the world easily due to continuous migration and traveling between countries and continents for different reasons such as business, tourism, politics, science, student exchange or to work. It is not possible to test everyone precisely to detect any diseases. We know that another pandemic is possible in the future due to microorganism mutation. Researchers should make continuous effort to improve the efficiency of protective equipment including face masks to ensure safety for people.

There has long been discussion about the connection between hospital hygiene and spreading of infection and the key role of face masks covering the mouth and nose to reduce health risk. After the COVID-19 pandemic, this discussion about the use of protective face masks, especially in the medicine care, is of high importance [3].

According to the standard EN 14,683 [4], protective masks are divided into three classes: I, II and IIR. These classes divide masks based on bacterial filtration efficiency, pressure differential and pressure resistance. Type II and IIR masks are dedicated mainly to medical staff. Currently, type II comprises three-layer protective masks made of properly selected polypropylene fibers, which limit the migration of pathogenic microorganisms through a nonwoven system. The masks effectively protect the user’s mouth and nose from contact with droplets and air that may contain infective microorganisms. Disposable masks reduce the possibility of transmitting infectious particles during person-to-person contact in both directions, because the three mask layers trap a higher amount of bacteria and viruses. One layer of the mask is an effective barrier against particles larger than 1 micron, while size of virus particles is usually between 0.06 and 0.14 microns, and they are sprayed by coughing and sneezing. Applying three layers to the mask structure improves filtration efficiency because the spaces between fibers are located randomly in each nonwoven, which means the microorganism transmission through all three layers is very difficult. Therefore, in this case, they also fulfill their role [5]; however, products made of 100% polypropylene have an adverse impact on the natural environment [6].

The production of polypropylene has a negative effect on the environment due to high energy consumption CO_2_ emission into the atmosphere, which adversely affects the climate on the earth [7]. Disposable masks after use become problematic waste that is not suitable for recycling. The improper waste management of disposable polypropylene masks results in the possibility of affecting living organisms, including food and animals [8,9]. Plastics can fill animals’ stomachs, reduce food intake and lead to starvation and even death. Most plastic waste, including disposable masks, pollute the soil environment, often in agricultural areas [9,10]. In addition, because of cultivation work, they are transferred from the surface to deeper layers of the soil, where they can be pushed to groundwater or reduce microbiological activity and affect the uptake of nutrients by plants [9,11].

Most plastics which originated on the market are made from the processing of fossil non-renewable raw materials, and the decomposition of finished products made of synthetic materials takes several hundred years. Plastics have been widely used for the last 60–70 years in many areas of the economy, causing significant increase in environmental pollutions. Plastic waste pollutes the seas and oceans to a significant extent. Considering that each person consumes about 130 kg of plastic goods, this means that approximately 30 kg of waste from each person could end up in the ocean [12]. These actions create serious economic and environmental problems. On the other hand, we are increasingly experiencing the problem of the amount of waste generated after the use of plastics. The market is currently dominated by products made of raw materials such as PE (polyethylene), PP (polypropylene), PET (polyethylene terephthalate), PS (polystyrene), and PVC (polyvinyl chloride), whose average decomposition period can be several hundred years [13]. Approximately 25 million tons of plastic waste are produced annually in EU countries, of which only one-third is recycled. Plastics production has skyrocketed in just a few decades—from 1.5 million tons in 1950 to 359 million tons in 2018 worldwide. Along with it, the amount of plastic waste has also increased. Although production fell diametrically in the first half of 2020 due to the COVID-19 pandemic, it recovered again in the second half of 2022 [14]. The European Union is tightening regulations on the use of plastic products, especially disposable food packaging. Activities in the field of environmental protection and ecology are a priority in the EU, and in recent years, numerous legislative measures have been developed that require transposition into national law. One of the most important problems is to reduce the consumption of plastic by entrepreneurs and consumers [13].

An alternative solution is to use reusable masks by sterilizing commonly used polypropylene fiber masks [15] or those made of, e.g., cotton fibers. The environmental impact of reusable masks depends on their type and how they are used. In an estimate based on annual mask consumption in the UK, Allison et al. [5] notes a similar environmental footprint between reusable masks and disposable surgical masks when considering the use of filters made of plastics and the hand laundering of reusable masks, but it is lower for reusable masks without filters and washing machines. Machine-washable reusable masks without filters result in higher water consumption than disposable masks due to the washing process. The literature reports that gas emissions during the production of reusable cotton fabric masks are near to those of surgical masks. However, the assessment of cotton fabric masks did not consider that a reusable cotton mask can be used about 180 times (without the use of disposable filters and including washing in the washing machine with ordinary clothes). In this case, the environmental footprint would be reduced [16]. However, the cumulative effects of washing contribute to material degradation [17]. In addition, reusable cotton masks without the use of plastic filters do not protect against the movement of viruses or bacteria.

Due to the growing problem of recycling plastic waste, a solution was proposed in the form of an innovative, disposable, partially biodegradable protective mask, which is the subject of cooperation between the Institute of Natural Fibers and Medicinal Plants National Research Institute and Genvita Sp. z o. o. In the proposed solution, the outer layer of the mask was replaced with a nonwoven made of natural fibers. This results in maintaining the filtering layer made of the existing polypropylene nonwovens. The third layer of the mask commonly made of PP fibers will be replaced with a layer made of 100% natural fibers: cotton and flax. This solution will allow the manufacture of a product that is approximately 50% biodegradable and safe for the natural environment and thus reduce the amount of synthetic fibers in disposable masks. Products made of 100% polypropylene are a huge burden on the natural environment [6]. Fibrous plants absorb CO_2_ from the atmosphere during vegetation and using solar energy produce their biomass, including substantial amounts of cellulose fibers [18,19,20]. An additional advantage of natural fibers such as flax fibers is their positive effect on the human body. Flax fibers are characterized by high hygroscopicity and air permeability, ensuring free breathing of the skin. This can eliminate the feeling of discomfort during moderate everyday physical effort thanks to high water retention. It has a positive effect on some physiological factors of the human body, reducing the rise of reactive oxygen species and oxidative stress. Linen products show a specific synergy with the skin, giving a pleasant feeling of cool touch and ensuring appropriate physiological comfort, guaranteeing the well-being of their user [21,22,23]. However, the nonwoven layer made of a mixture of natural fibers (cotton/flax) has no affinity with the polypropylene layers of the mask. It is important to maintain the possibility of linking individual layers of the mask with each other using the ultrasound method used for the currently produced masks. The problem that arises is the binding of the natural nonwoven layer with polypropylene. To achieve the intended results, a welding process of all layers of the mask will be developed. Natural fibers do not melt at the melting temperature of polypropylene, which prevents the process of welding polypropylene nonwovens with natural nonwovens using ultrasound. The solution to this problem is to cover the edge of the nonwoven fabric made of natural fibers with a layer of a polymer film using a hot-pressing process. However, the use of materials such as PE, PP, PET, PS and PVC as polymer film may also have a negative impact on the environment. Therefore, a film made of biodegradable polymers, such as polylactic acid (PLA), which is one of the leading bioplastics on the market [24,25], will be used in the study.

The goal of the current study was to develop a natural flax/cotton nonwoven layer in with appropriate properties to replace the outer PP layer of face mask type II as well as the method of mask layers connection to meet the challenge of non-biodegradable waste reduction.

## 2. Materials and Methods

### 2.1. Materials

Flax and cotton fibers were used to develop the nonwoven layer dedicated to replacing one of the polypropylene layers of disposal face masks.

**Figure 1:**
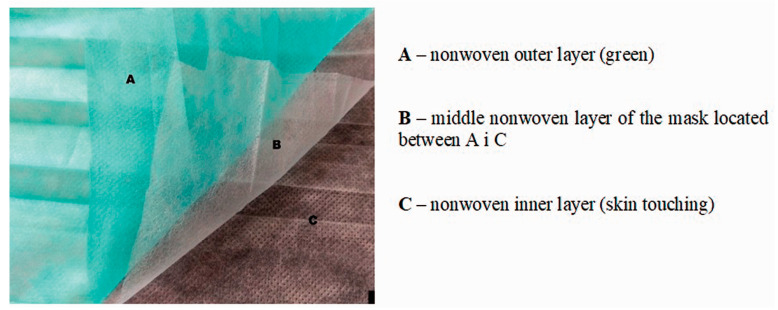
The three polypropylene nonwoven layers of mask type II. A: nonwoven outer layer (green); B: middle nonwoven layer of the mask located between A and C; C nonwoven inner layer (skin touching). Fot. Kicińska-Jakubowska, A. [15].

The type II face mask consists of three layers, as shown in Figure 1. In the assumption of the conducted experiment, the innovative developed natural layer is intended to replace the outer layer (layer A in Figure 1) of the mask.

The nonwoven was produced from cottonized flax and cotton fibers with use of the *Spunlace* water needling method. Three different fiber compositions were applied to the nonwoven manufacture:

- 10/90 bleached cottonized flax/cotton;
- 20/80 bleached cottonized flax/cotton;
- 30/70 bleached cottonized flax/cotton.

#### 2.1.1. Fibers’ Preparation

High-quality flax fibers were selected for further processing in order to obtain fibers with physical properties such as length, linear density and lack of impurities, which are suitable to mix with cotton fibers. The fibers’ mechanical modifications were carried out according to cottonization technology, which consists of the following operations: cleaning, shortening, and dividing glued fibers to smaller complexes. The cottonization of the flax fibers was conducted with use of a carding device so-called RCz. The mechanical process led to shortening of the fibers and increasing the divisibility of their complexes by their longitudinal division due to partial destruction of the middle plates and longitudinal connections of the fiber bundles [26]. The obtained cottonized flax fibers were characterized by an average linear density of 0.4 tex, length of 34 mm, and an impurities content of 0.5%.

Cottonized flax was bleached at a subsequent stage to give softness, delicateness and white color to the fibers.

The fibers were treated by an alkali bath to bleach them according to a conventional process. The bleached cottonized fibers showed the following values of basing parameters: average linear density 0.3 tex, length 25 mm, and impurities content at the level of 0.3%. Commercially available cotton fibers used to prepare blended nonwovens were characterized by an average linear density of 0.1 tex, a length of 12 mm and an impurities content of 0.1%.

Cottonized bleached flax fibers and cotton fibers were mixed with planned proportions with the use of a carding machine.

#### 2.1.2. Development and Production of Nonwovens

**Figure 2:**
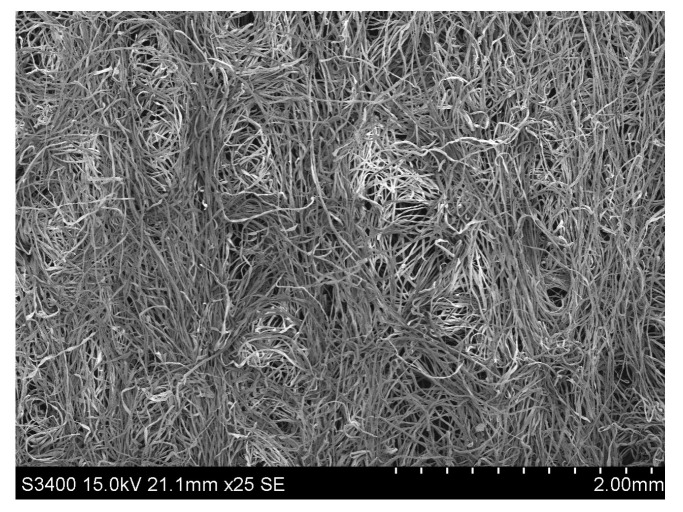
SEM image of surface of 10/90 bleached cottonized flax/cotton nonwoven.

**Figure 3:**
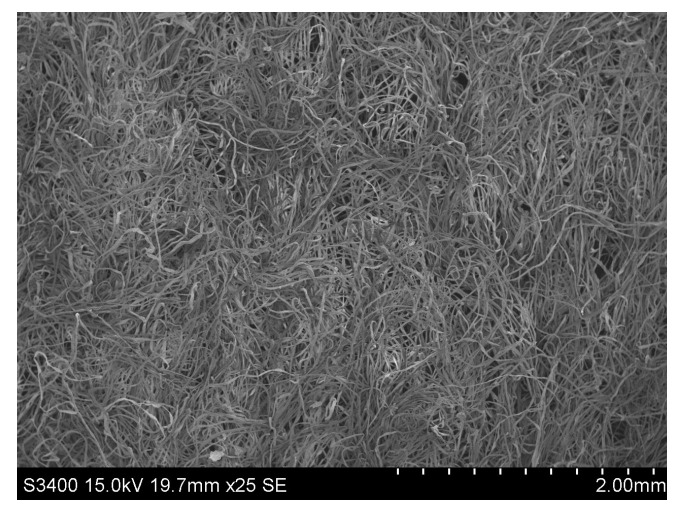
SEM image of surface of 20/80 bleached cottonized flax/cotton nonwoven.

**Figure 4:**
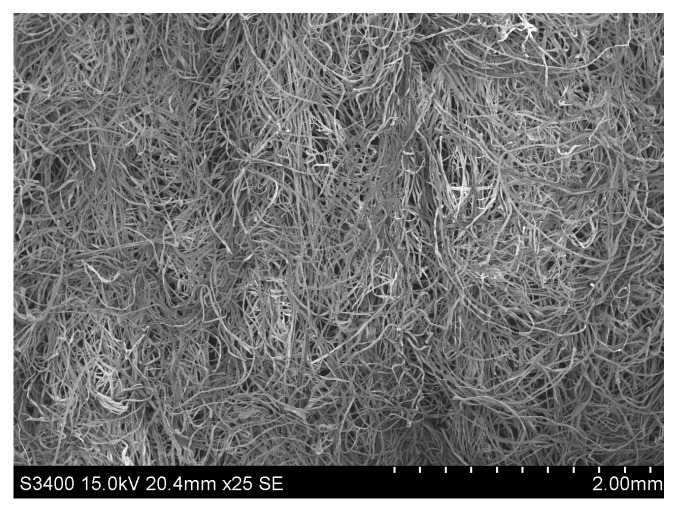
SEM image of surface of 30/70 bleached cottonized flax/cotton nonwoven.

The blended flax/cotton nonwovens were produced with use of the Spunlance method. The Sunplance method is the process of hydroentangling that involves the use of water needles to entangle the fibers. As a result of needling, under the action of water interweaving needles, the fibers move in the direction transverse to the plane of the fleece and in this way bind individual layers of the fleece [27]. This process leads to a three-dimensional structure of the fleece and contributes to the density of the fleece (Figure 2, Figure 3 and Figure 4).

#### 2.1.3. Polymer Film Application

The polypropylene face mask layers were glued together with the flax/cotton layer with the use of the developed method based on the selected polymer film. To select the most effective layers connection, the following polymer films were applied:

- polypropylene PP film with a thickness of 0.035 mm;
- polylactide PLA film with a thickness of 0.025 mm;
- polylactide PLA film with a thickness of 0.050 mm.

The experiments covered the use of two selected polymers: PP and PLA. PP was used for preliminary studies, because the film can be applied for a wide range of thicknesses. The aim of the first stage of the research was to select the polymer film deposition parameters, which ensure effective connection of the nonwoven layers. Nevertheless, to obtain a fully biodegradable layer, the PP film was replaced with PLA film at the last stage of the study.

For preparation of the polymer films, the welding process was carried out using heating plates with manual pressure and temperature control. Directly before the welding process, a pointwise temperature measurement was carried out on the surface of the plate. The welding temperature, depending on the type and thickness of the polymer films, was in the range of 190–230 °C, the pressure force was 10 kPa and the application time was 10–20 s.

Therefore, since the film is only an element of binding the natural layer with the PP layer of the mask, in the continuous technological process, the film will be inserted only on the edges of the mask. However, to check the effect of the film on the structural and mechanical properties of the blended nonwovens, the films were applied to the entire surface of the samples.

### 2.2. Methods

The blended nonwovens and blended nonwovens with applied polymer films were tested according to appropriate standards to evaluate the structural and mechanical parameters:

Surface density (g/m^2^) in accordance with EN 29073-1:1994 [28]. The tests were performed using a Mettler PM 480 electronic balance (Mettler Toledo, Greifensee, Switzerland).

Thickness (mm) in accordance with EN ISO 9073-2:2002 [29]. The tests were performed using a J-40-V electronic thickness gauge (Checkline, Bad Bentheim, Germany).

Breaking force (N) and elongation (%) in accordance with EN 29073-3:1994 [30]. The tests were performed using a STATIMAT ME automatic tensile tester (Textechno, Mönchengladbach, Germany).

Stiffness (mNm) in accordance with EN ISO 9073-7:2011 [31]. The tests were performed using an SDL 003B stiffness testing device (SDL Atlas, Manchester, UK).

Angle of recovery (°) in accordance with EN ISO 2313-2:2021-12 [32]. The tests were performed using a Montsant MR-1 wrinkle recovery tester (Sossna GmbH, Dorsten, Germany).

The statistical evaluation of the test results was performed on the ANOVA test at a significance level of 0.05 using the Statistica software 8.0 (StatSoft Polska Sp. z o.o., Kraków, Poland).

In addition, for blended nonwovens, the following parameters were determined.

Air permeability (mm/s) in accordance with EN ISO 9237:1998 [33]. The tests were carried out using the Air Permeability Tester III FX 3300 apparatus (Textest Instruments, Schwerzenbach, Switzerland).

Thermal resistance (m^2^K/W) and water vapor resistance (m^2^Pa/W) in accordance with EN ISO 11092:2014-11 [34]. The tests were carried out using a heat-insulated SGHP-8.2 sweating plate with a CEO 910-4 environmental chamber (Measurement Technologiec Inc., Houma, LA, USA).

Hygroscopicity 65% and 100% (%) in accordance with PN-P-04635:1980 [35].

Ability to water sorption (drop method) (s) in accordance with JIS 1090:1990 [36]. The tests were carried out using a station for testing the sorption of textiles consisting of a pipette rim for attaching textiles (INF&MP, Poznań, Poland).

Cover ratio analysis of the surface of 0.24 mm^2^ of every consecutive layer of the mask. The analysis was performed on a Nikon Eclipse E400 optic microscope (Nikon, Tokyo, Japan) with an integrated camera by which a series of photographs was taken and a software specialist NIS-Elements BR (Nikon, Tokyo, Japan) by which a surface area of the sample and of the free space in each sample was determined. In the next step, a cover ratio was determined.

Biodegradation tests were conducted to determine the damage after specified incubation conditions of the developed natural nonwovens dedicated for use in masks and nonwovens covered by polymer films. The tests were carried out in accordance with ISO 846:2019 Plastics—Evaluation of the action of microorganisms by exposing the materials due to the action of soil microorganisms [37]. The test consisted of placing samples of nonwoven fabrics in jars filled with biologically active soil for 11 weeks. The reference samples were strips of raw bleached cotton fabric with a weight of 250 g/m^2^ mass per square, whose strength after the action of soil microorganisms dropped below 25% of the initial strength required by the standard. Two samples of the tested nonwovens and one sample of cotton fabric as a reference were placed in each jar. The test was performed in triplicate.

## 3. Results

**Figure 5:**
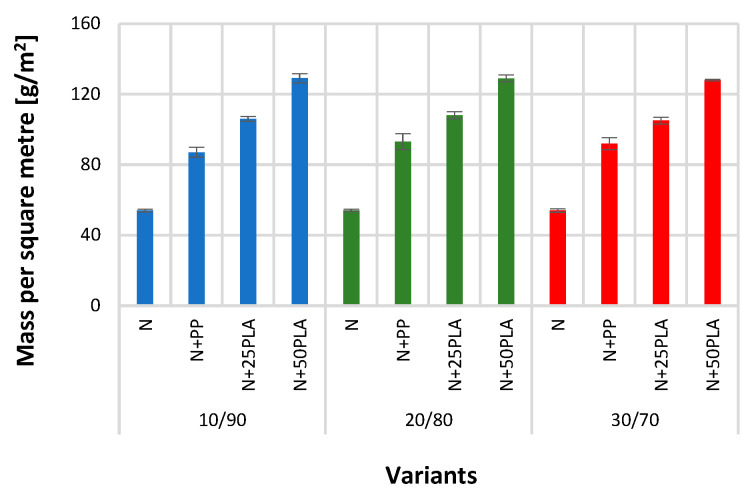
The results of test of mass per square meter of the analyzed variants of nonwovens and nonwovens with PP and PLA films.

**Figure 6:**
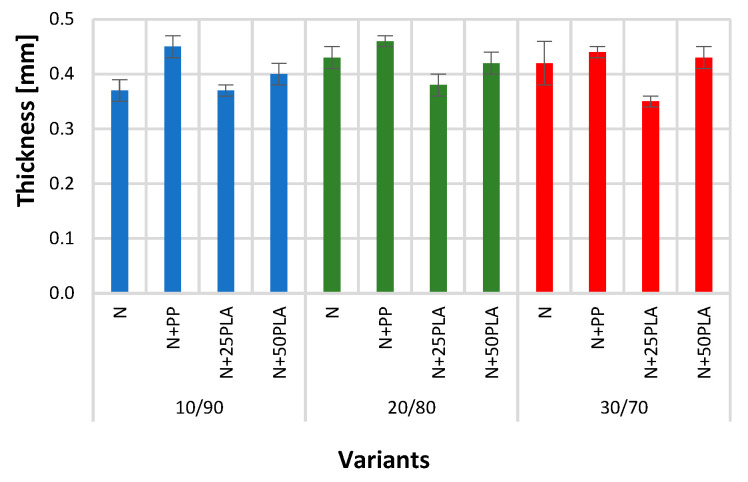
The results of test of thickness of the analyzed variants of nonwovens and nonwovens with PP and PLA films.

**Figure 7:**
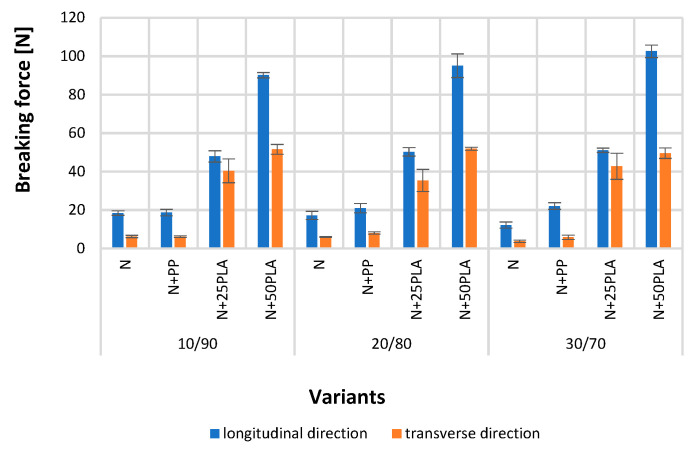
The results of breaking force test of the analyzed variants of nonwovens and nonwovens with PP and PLA films.

**Figure 8:**
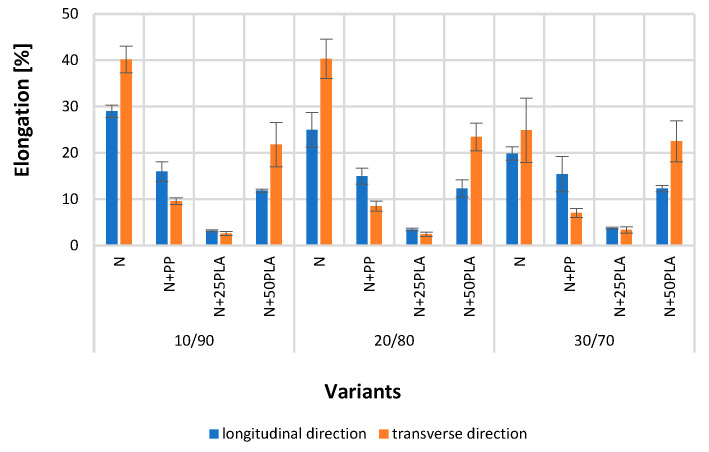
The results of elongation test of the analyzed variants of nonwovens and nonwovens with PP and PLA films.

**Figure 9:**
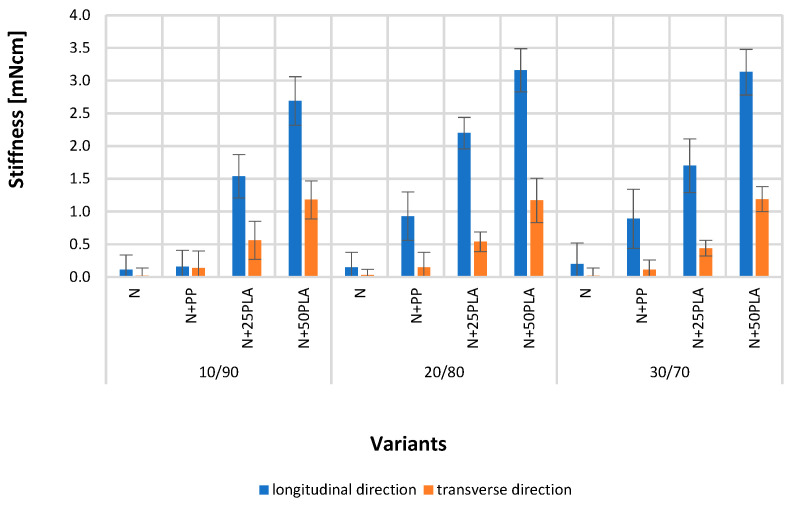
The results of stiffness test of the analyzed variants of nonwovens and nonwovens with PP and PLA films.

**Figure 10:**
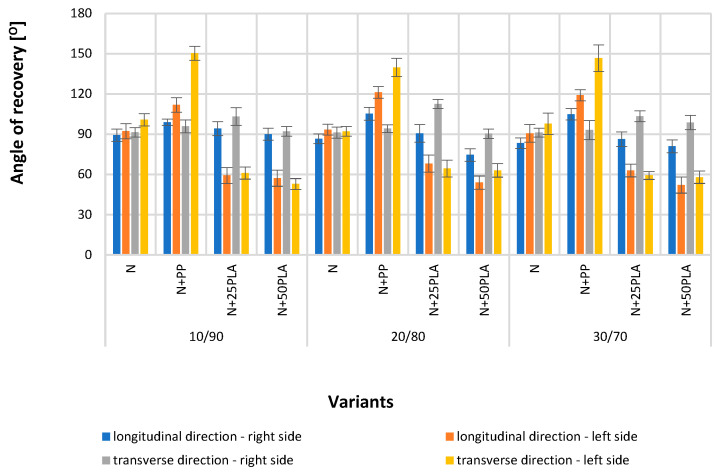
The results of angle of recovery test of the analyzed variants of nonwovens and nonwovens with PP and PLA films.

Figure 5, Figure 6, Figure 7, Figure 8, Figure 9 and Figure 10 present the results of metrological tests of nonwovens without film and with PP and PLA films with use of the following symbols:
**Symbol****Description of Sample**NFlax/cotton nonwovenN+PPFlax/cotton nonwoven covered by PP filmN+25PLAFlax/cotton nonwoven covered by PLA film with a thickness of 0.025 mmN+50PLAFlax/cotton nonwoven covered by PLA film with a thickness of 0.050 mm

### 3.1. Analysis of Structural Parameters

The obtained nonwovens showed the similar weight (mass per square meter); the weight values of each nonwoven (N) composition were in range 51–54 g/m^2^. Statistical analysis showed no significant differences between mass per square meter of 10/90 and 20/80 nonwovens. As a result of applying a polymer film, the weight of nonwovens covered with a polypropylene film (N+PP) increased by approximately 40%, while in the case of nonwovens covered with a polylactide film, for the film thickness of 0.025 mm (N+25PLA), the weight increased 50%, and for the thickness of 0.050 mm (N+50PLA), it increased 60% (Figure 5). This is due to the weight of the polymer used [38,39]. All differences between mass per square meter of each nonwoven and their variants with applied PP and PLA films were statistically significant.

The thickness of all blended nonwovens without films (N) is in the range of 0.37–0.43 mm. Nonwovens covered with a 0.025 mm PP film (N+PP) and a 0.050 mm PLA (N+50PLA) film are slightly thicker than nonwovens without a coating because they have a thickness of 0.40–0.46 mm. In the case of nonwovens covered with PLA 0.025 mm film (N+25PLA), the thickness was 0.35–0.38 mm (Figure 6), wherein there were no significant differences between the thickness of all tested variants.

### 3.2. Analysis of Mechanical Properties

The values of breaking force of developed nonwovens (N) tested in the longitudinal direction indicated the parameters’ dependance on the percentage of flax/cotton fibers content in the blend: 10/90–18.42 N, 20/80–17.25 N and 30/70–12.19 N, wherein there were no significant differences between results of breaking force tests of 10/90 and 20/80 variants. The breaking strength therefore decreased with increasing the proportion of cottonized bleached flax fibers in the nonwovens, because the bleaching process can lead to moderately weakened flax fibers [40]. In the transverse direction, values of nonwoven breaking force depended on the percentage share of flax and cotton fibers: 10/90–6.28 N, 20/80–5.99 N and 30/70–3.79 N, wherein there were no significant differences between the results of the breaking force in case of 10/90 and 20/80 variants. Lower values of breaking force tested in the transverse direction in comparison to the longitudinal direction are a predictable phenomenon for nonwovens with the structure of blended nonwovens. The derived blended nonwovens have an orthotropic orientation, thanks to which much higher strength is obtained in the direction of fleece formation than in the perpendicular direction [41].

Blended nonwovens covered with both polypropylene (N+PP) and polylactide films (N+25PLA, N+50PLA) are characterized by higher breaking force compared to nonwovens without the film. The breaking force of all types of nonwovens covered with a 0.035 mm PP film reached values of 18.72–22.09 N in the longitudinal direction, but statistically significant differences were noticed only for composition 30/70. In case of tests conducted in the transverse direction, the results were in range of 5.84–8.07 N, and the differences between results were not statistically significant for all variants. Nonwovens covered with a 0.025 mm PLA film, compared with nonwovens without film, showed statistically significant differences in values of breaking force, which were in the range of 47.93–51.12 N for the longitudinal direction test and 35.39–42.77 N for the transverse direction. Nonwovens covered with a 0.050 mm PLA film obtained the highest breaking force, which significantly differed from other ones and reached values from 90.17 to 102.53 N in the longitudinal direction and 49.62 to 51.90 N in the transverse direction (Figure 7).

The combination of polymer films with nonwoven fabrics contributed to the strengthening of the obtained intermediates. The polymer films penetrate the internal structure of the nonwoven and weld its randomly laid fibers so that the forces acting are transmitted through both the nonwoven fibers and the polymer layers [39,42].

The film applied to the blended nonwovens caused a reduction in the values of elongation at maximum force. The elongation of nonwovens with the film in the longitudinal direction is in the following ranges: 19.88–24.99% for blended nonwovens (N), 14.98–15.59% for blended nonwovens with a 0.035 mm PP film (N+PP), 11.87–12.35% for blended nonwovens with a 0.050 mm PLA film (N+50PLA), and 3.17–3.76% for blended nonwovens with a 0.025 mm PLA film (N+25PLA). The differences of values of elongation for each nonwoven and their variants with PP and PLA film coating were statistically significant.

In the transverse direction, elongation was lower in comparison to values tested in the longitudinal direction and reached values in the following ranges: 24.92–40.34% for blended nonwovens (however, there were no significant differences between the elongation of nonwovens with fibers composition 10/90 and 20/80), and 21.79–23.44% for blended nonwovens with a 0.050 mm PLA film. The differences between the elongation of nonwovens 30/70 without films and with applied film 0.050 mm PLA were not statistically significant. An elongation of 7.05–9.58% was obtained for mixed nonwovens with 0.035 mm PP film and 2.42–3.36% for mixed nonwovens with 0.025 mm PLA film (Figure 8). The lowest values of elongation in both tested directions were observed in the case of all nonwoven’s compositions covered by PLA film with a thickness of 0.025 mm; however, the highest values of elongation were shown by nonwovens without any films. Reducing the elongation of blended nonwovens reinforced with polymer films as bonding layers will be a positive factor improving the mask quality, which contains natural flax/cotton nonwoven as one of the three layers [43].

### 3.3. Analysis of Physical Properties

All types of flax/cotton nonwovens (N) are characterized by lower stiffness tested in the longitudinal direction, where the values were in range: 0.11–0.20 mNcm in comparison to the values tested in transverse direction, which showed values between 0.02 and 0.03 mNcm (Figure 9), but the differences were not statistically significant. The commencement of polymer films (N+PP, N+25PLA, N+50PLA) on the nonwovens surface caused an increase in the stiffness, especially in the longitudinal direction. To ensure the high durability of the binding of the nonwoven with the film, the process of applying polymer films was carried out under high temperature and pressure conditions, which contributed to their stiffening [39,44].

The test of the nonwovens (N) angle of recovery indicated that the values were within limits 83.30–100.70° and no statistically significant differences were noticed between the angle recovery values of all the nonwoven variants.

Wu et al. proved that PLA film has a weak formability [45]. Nonwovens covered with PLA films (N+25PLA, N+50PLA) reached the lowest values of the angle of recovery in comparison to other tested variants. Polymer films were deposited on the left side of the nonwoven; for this reason, nonwovens covered with PLA film were tested on the left side. The tests showed statistically significant differences between the angle of recovery of nonwovens with and without PLA film; the values were in the range of 52.10–68.0° in the longitudinal direction as well as 52.80–64.30° in the transverse direction (Figure 10). Low formability can be beneficial for the final product because it allows keeping the mask in its preliminary shape.

### 3.4. Analysis of Biophysical Parameters

Flax/cotton nonwovens are characterized by high air permeability at the level from 1196 to 1343 mm/s, while the air permeability of the nonwoven increased significantly when the share of bleached cottonized flax fibers in the nonwoven increased (Table 1). A high level of the air permeability is an advantage of the nonwovens because it can influence and improve the breathability of the final product for both skin and basic breathing. This contributes to maintaining thermal balance and the removal of carbon dioxide from the nose/mouth/skin area through the mask layers to the outside [46].

The water vapor resistance of the textiles determines their capacity to transfer heat in the form of sweat at any level of humidity from the skin [47]. The water vapor resistance test of the flax/hemp nonwoven did not show statistically significant differences between all variants; the values were in range from 3.3119 to 4.6196 m^2^Pa/W. The results indicated the high capacity of the developed nonwovens for moisture removal, which can have an effect on improving the comfort of the face mask wearing [48].

The differences of results of thermal resistance between all tested nonwovens are statistically significant; the values were in range 0.0428–0.0470 m^2^K/W. The thermal resistance values of flax/cotton nonwovens corresponded to values recommended for light, thin textiles [49].

### 3.5. Analysis of Sorption Properties

The hygroscopicity of the all blended nonwovens tested at 65% relative humidity of air were in the range 7.10–7.64%. In test conditions of 100% relative humidity of air, the value of hygroscopicity increased with the increase in flax fibers share in the nonwovens, wherein the differences between variants of nonwovens were not statistically significant.

The wettability of flax/cotton nonwovens was evaluated with use of the drop method by the measurement of time needed to complete water drop absorption at the nonwoven surface [50]. The time needed to complete water drop absorption by the tested nonwovens was in the range 0.2–0.4 s. The wettability of variants of nonwovens was not statistically significant.

### 3.6. Cover Ratio Analysis of the Surface

The test results of the nonwovens cover ratio with fibers showed that the filling decreased with the increase in the amount of cottonized flax fibers in the nonwovens. However, for nonwovens with a content 10% and 20% of cottonized flax fibers, the ratio of surface filling was appropriately 99.6% and 99.1%. For a 30/70 flax/cotton nonwoven, the filling with fibers in the nonwovens was 98.9%. Nevertheless, the differences between values of cover ratio of all tested nonwovens variants were statistically insignificant.

This means that the developed structures of blended nonwovens can help support the other layers of the mask in terms of filtration efficiency [15].

### 3.7. Biodegradability Results

**Figure 11:**
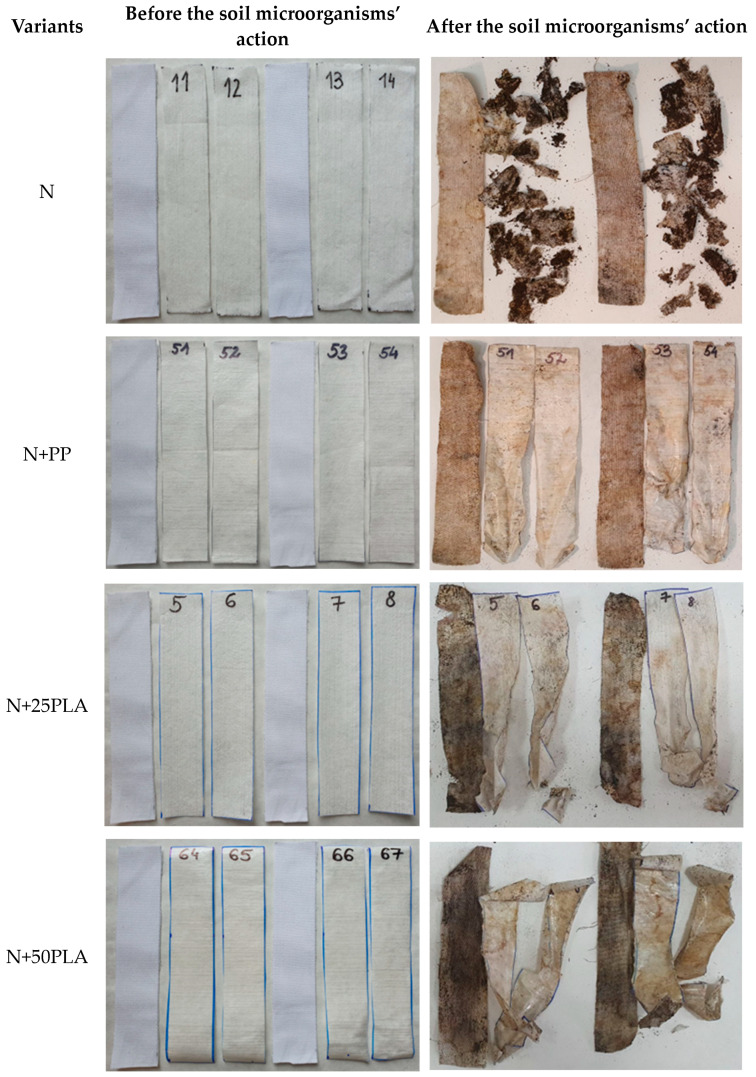
Images of nonwoven samples before and after the soil microorganisms’ action.

**Figure 12:**
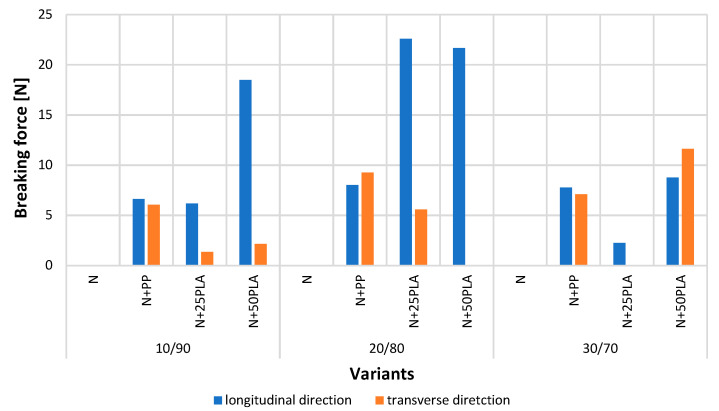
The test results of breaking force of nonwovens without film and with PP and PLA films after the action of soil microorganisms.

Figure 11 presents the example of nonwoven samples before and after the action of soil microorganisms. Figure 12 presents the results of strength properties of nonwovens without film and with PP and PLA films before and after the action of soil microorganisms.

The samples of all types of flax/cotton nonwovens without any films (N) applied were biodeteriorated. The samples’ decomposition process caused by the action of soil microorganisms was monitored for 11 weeks. After this period, the changes of color and the samples’ structure were advanced, which prevented nonwoven breaking force measurement (Figure 11).

Cotton fabric was used as a reference sample. The action of soil microorganisms caused a reduction in the tensile strength of the cotton sample. The breaking force decreased from 168.36 N to an average value of 21.96 N, which is 12.88% of the initial strength. The percentage decrease of the strength (12.88%) allows us to conclude that the tested sample was biodeteriorated by soil microorganisms within 11 weeks according to the Standard ISO 846:2019 [37].

Nonwovens with PP film (N+PP) after the action of soil microorganisms were extracted from the soil in their entirety, but their color had changed. Their tensile strength tested in the longitudinal direction showed lower values (Figure 12) compared to previous results (Figure 7). The largest decrease in the breaking force was observed in the case of the nonwoven with a fiber share of 30/70, where the value had decreased from 22.09 to 7.78 N. However, in the case of the transverse direction, the breaking force value slightly decreased or increased; the most significant growth from 5.84 to 7.10 N was noticed for the nonwoven with a 30/70 fiber composition.

The samples of nonwovens covered with a PLA films (N+25PLA, N+50PLA) were extracted from the soil in their entirety, and the color was also changed, and their shape had been deformed. The samples were significantly weakened by the action of soil microorganisms. The shapes of most of the samples enabled performing the tests; however the values of breaking force were low. The biggest decrease from 102.53 to 8.76 N was recorded for the 30/70 variant covered with 0.050 mm PLA film. In the case of the 20/80 variant, covered with 0.050 mm PLA, and the 30/70 variant, covered with 0.025 mm PLA, the samples were so attenuated that the results could not be registered by the tensile strength device.

## 4. Conclusions

The results of this study confirmed that the developed flax/cotton nonwoven can replace one of three polypropylene layers of face masks type II in order to reduce negative masks’ waste effect on the environment.

All the analyzed blended nonwovens made of bleached cottonized flax fibers (i.e., 10/90, 20/80 and 30/70 flax/cotton) are characterized by an appropriate structure to be applied for unprecedented use as one layer of a protective face mask. The developed natural face mask layer has a high cover ratio, which can support the PP filter layers in effective filtration. The flax/cotton nonwoven has potential to improve nose/mouth and skin breathing during mask wearing thanks to its high air permeability, water vapor and thermal resistance.

The nonwoven with a composition of 10% of flax and 90% of cotton fibers is characterized by the highest cover ratio, which means that its filtration ability is higher in comparison to other 20/80 and 30/70 nonwovens. Taking into account the barrier properties against microorganisms, the 10/90 flax/cotton nonwoven should be recommended to replace the polypropylene layer in the face mask. The flax/cotton 20/80 nonwoven shows also properties suitable for mask production, and its cover ratio is higher than 90%. In both cases, the economic aspects and easy-going technological process indicate that flax fibers with 10% and 20% shares in natural fiber nonwovens can be consider as promising in future work.

The application of biodegradable films to glue the nonwoven layers will not only contribute to the effective connection of the natural layer with the filtering PP layers but also contribute to strengthening the nonwoven, resulting in an increase in stiffness and reducing deformations, which will support maintaining the proper shape of the mask during its use.

The developed semi-finished product, e.g., a flax/cotton nonwoven with PLA film applied to its surface, will contribute to improving the ecological aspects of the protective mask. The blended flax/cotton nonwovens were completely degraded after 11 weeks of conditioning in appropriate degradation conditions. At the same time, the PLA film was significantly degraded, and the structures of the samples were seriously distorted, which led to the weakening of the structure of the nonwoven covered by the film.

The developed product in the form of a flax/cotton nonwoven with a connecting element, consisting of biodegradable polymer films, is ready to be implemented in mask production. It is worth emphasizing that replacing the outer PP layer of the face mask type II with a biodegradable one made of flax/cotton nonwoven enriched with a biodegradable PLA film to glue the layers together has the potential to improve the environmental performance of the mask and face the challenge of protecting users against harmful microorganisms.

## Figures and Tables

**Table 1:** Result of biophysical parameters tests of nonwovens without films. Results are expressed as mean and standard deviation (SD). Values sharing the same letter are not significantly different from each other (Tukey’s HSD, *p* < 0.05).

Parameter			Variants		
			10/90	20/80	30/70
Air permeability	AV	mm/s	1196	1268	1343
	SD		53	63	68
Thermal resistant	AV	m^2^K/W	0.0446	0.0470	0.0428
	SD		0.0065	0.003	0.004
Water vapor resistant	AV	m^2^Pa/W	4.6196 ^a^	3.7773 ^a,b^	3.3119 ^b^
	SD		0.2968	0.3898	0.1646
Hygroscopicity at 65%	AV	%	7.48	7.64	7.10
	SD		0.2	0.21	0.36
Hygroscopicity at 100%	AV	%	14.46 ^a^	14.82 ^a,b^	15.42 ^b^
	SD		0.31	0.38	0.24
Water sorption	AV	s	0.2 ^a^	0.3 ^a,b^	0.4 ^b^
	SD		0.1	0.1	0.2
Cover ratio	AV	%	99.6 ^a^	99.1 ^a,b^	98.9 ^b^
	SD		3.1	3.0	3.0

Note: In Table 1, sign: ^a,b^—represents the groups for which the mean values do not differ statistically at the assumed significance level. The mean values labeled with the same letter (a or b) do not differ statistically at (α = 0,05).

## Data Availability

Not applicable.
